# Altered Responses to Homeostatic Cytokines in Patients with Idiopathic CD4 Lymphocytopenia

**DOI:** 10.1371/journal.pone.0055570

**Published:** 2013-01-30

**Authors:** Florence Bugault, Daniela Benati, Luc Mouthon, Ivan Landires, Pierre Rohrlich, Vincent Pestre, Jacques Thèze, Olivier Lortholary, Lisa A. Chakrabarti

**Affiliations:** 1 Unité d'Immunogénétique Cellulaire, Institut Pasteur, Paris, France; 2 Université Paris Descartes, Pôle de Médecine Interne, Hôpital Cochin, Assistance Publique-Hôpitaux de Paris (AP-HP), Paris, France; 3 INSERM U645, Besançon, France; 4 Université de Besançon, Besançon, France; 5 Service de Pédiatrie, Centre Hospitalo-Universitaire de Besançon, Besançon, France; 6 Unité de Mycologie Moléculaire, Institut Pasteur, Paris, France; 7 CNRS URA 3012, Paris, France; 8 Université Paris Descartes, Service des Maladies Infectieuses et Tropicales, Centre d'Infectiologie Necker-Pasteur, Hôpital Necker-Enfants Malades, AP-HP, Paris, France; Rush University, United States of America

## Abstract

Idiopathic CD4 lymphocytopenia (ICL) is a rare immune deficiency characterized by a protracted CD4^+^ T cell loss of unknown etiology and by the occurrence of opportunistic infections similar to those seen in AIDS. We investigated whether a defect in responses to cytokines that control CD4^+^ T cell homeostasis could play a role in ICL. Immunophenotype and signaling responses to interleukin-7 (IL-7), IL-2, and thymic stromal lymphopoietin (TSLP) were analyzed by flow cytometry in CD4^+^ T cells from 15 ICL patients and 15 healthy blood donors. The induction of phospho-STAT5 after IL-7 stimulation was decreased in memory CD4^+^ T cells of some ICL patients, which correlated with a decreased expression of the IL-7Rα receptor chain (R = 0.74, p<0.005) and with lower CD4^+^ T cell counts (R = 0.69, p<0.005). IL-2 responses were also impaired, both in the Treg and conventional memory subsets. Decreased IL-2 responses correlated with decreased IL-7 responses (R = 0.75, p<0.005), pointing to combined defects that may significantly perturb CD4^+^ T cell homeostasis in a subset of ICL patients. Unexpectedly, responses to the IL-7-related cytokine TSLP were increased in ICL patients, while they remained barely detectable in healthy controls. TSLP responses correlated inversely with IL-7 responses (R = −0.41; p<0.05), suggesting a cross-regulation between the two cytokine systems. In conclusion, IL-7 and IL-2 signaling are impaired in ICL, which may account for the loss of CD4^+^ T cell homeostasis. Increased TSLP responses point to a compensatory homeostatic mechanism that may mitigate defects in γc cytokine responses.

## Introduction

Idiopathic CD4^+^ lymphocytopenia (ICL) is an immune deficiency characterized by persistently decreased CD4^+^ T lymphocyte numbers in the absence of HIV infection or other known causes of T cell depletion [1]–[8]. Clinical signs are variable, with a subset of patients presenting with life threatening opportunistic infections very similar to those seen in AIDS, including *Cryptococcus neoformans* meningitis, disseminated *Mycobacterium avium* infection, tuberculosis, and *Pneumocytis jiroveci* pneumonia [5], [9]. ICL is most frequently diagnosed in adults [1], though cases have also been reported in children [6]–[8]. Studies launched in the early 90's to identify a possible retrovirus associated with ICL were inconclusive [1] and the etiology of ICL remains unknown in most cases.

Mechanistic studies of T cell function in ICL have provided evidence for increased immune activation and increased susceptibility to apoptosis, in a process that is partially dependent on Fas expression [10], [11]. Abnormal immune activation was confirmed by the detection of an increased T cell turnover [12] and by the presence of microbial translocation products in the plasma of ICL patients, similar to findings reported in HIV infection [13]. Another factor that may contribute to the loss of CD4^+^ T cells is a decreased clonogenic capacity of the bone marrow in ICL patients [14]. A hypomorphic mutation of the recombination activating gene 1 (RAG1), which triggers TCR and immunoglobulin gene rearrangements, was recently identified in a child with ICL [15]. Thus, the spectrum of immune defects associated with RAG mutations may include ICL in addition to Omenn syndrome, granulomatous disease, and severe combined immunodeficiency [16]. A decrease in p56 Lck activity was initially reported in one ICL case, raising the possibility of defective TCR signal transduction [17]. This notion was recently supported by the identification of mutations that impair but do not abrogate TCR signaling in some ICL patients. The mutations targets the adaptor protein uncoordinated 119 (UNC119), which is required for Lck transport and activation [18], or the magnesium transporter 1 (MAGT1), which contributes to the proper activation of phospholipase C gamma 1 (PLCγ1) [19]. It should be noted, however, that only a few of ICL patients show signs of impaired TCR signaling [20].

Depletion of the CD8^+^ T cell population occurs in a subset of ICL patients and is associated with more severe disease outcome than CD4^+^ T cell depletion alone [12]. The B cell compartment also shows abnormalities, including an accumulation of immature/transitional B cells that may be driven by increased levels of IL-7 in peripheral blood [21]. However, circulating immunoglobulin levels usually remain in the normal range, and the spectrum of opportunistic infections is indicative of T cell rather than B cell immunodeficiency. The increase of circulating IL-7 in ICL patients parallels that seen in HIV-infected patients with severely depleted CD4^+^ T cell counts [22], [23], and likely reflects a compensatory mechanism that promotes homeostatic T cell proliferation in response to lymphopenia. Increased availability of IL-7 is thought to result from lower consumption of the cytokine by a reduced T cell pool [22], [24]. In addition, more recent evidence suggest that lymphopenia also triggers an increased production of IL-7 by stromal cells in the thymus [25] and possibly the bone marrow [26]. Raised IL-7 concentration can then facilitate T cell proliferation in response to self and non-self antigens [24]. However, increased IL-7 levels do not appear sufficient to restore the CD4^+^ T cell counts in ICL, raising the possibility of downstream defects in the IL-7 response pathway. Another indication that cytokines may be involved in ICL pathogenesis arises from reports of a beneficial effect of IL-2 immunotherapy. Case reports suggested that recombinant IL-2 administration could raise CD4^+^ T cell counts in ICL patients and help improve the clinical outcome of opportunistic infections, including cryptococcal meningitis [6], chronic mycobacterial disease [27], [28], and relapsing herpes zoster infection [29]. Recently, we reported that IL-2 administration improved CD4^+^ T cell counts in three out of four treated patients [20]. In the course of this study, we noted a markedly decreased expression of the chemokine receptor CXCR4 at the surface of CD4^+^ T cells from ICL patients, suggesting that impaired CXCR4-dependent cell trafficking may contribute to the immunodeficiency. Importantly, CXCR4 expression was normalized in the three patients who responded to IL-2 therapy but not in the non-responder. Given the known capacity of γc cytokines to upregulate CXCR4 expression [30]–[32], one hypothesis is that defective cytokine responses lead to decreased chemokine receptor expression in ICL.

To explore the involvement of cytokines in ICL pathogenesis, we tested the capacity of ICL patient cells to respond to IL-7 and IL-2, two key γc family cytokines known to control the CD4^+^ T cell pool size [33]. We also measured responses to TSLP, a cytokine that shares structural and functional properties with IL-7, and whose role in human T cell homeostasis remains incompletely understood [34], [35]. We found that CD4^+^ T cells from ICL patients responded less efficiently to both IL-2 and IL-7. The two defects correlated together, suggesting they may synergize in perturbing CD4^+^ T cell homeostasis. Intriguingly, responses to TSLP were increased in CD4^+^ T cells of ICL patients, revealing a compensatory role for this IL-7-like cytokine.

## Methods

### Study design

All patients met the definition of ICL established by the Center for Disease Control, namely the occurrence of a CD4^+^ T cell count <300 mm3 or <20% of lymphocytes on at least two separate measurements [1]. All patients were seronegative for HIV-1, HIV-2, HTLV-1, HTLV-2, HBV, HCV, and HHV-8. HIV load was undetectable in all patients and none of them had clinical or biological evidences of active EBV infection, systemic lupus erythematosus, Sjögren syndrome, or sarcoidosis. ICL diagnosis was confirmed after ruling out known causes of immunodeficiency. Clinical and immunological characteristics of the ICL patients included in the study are reported in Table 1.

**Table 1:** Clinical and imunological charateristics of ICL patients.

Patient ID	gender	age (yrs)	T CD4/mm3 at diagnosis	disease duration (months)	Lympho/mm3	T CD4/mm3	T CD8/mm3	opportunistic infections
P1	F	27	42	44	580	47	220	Human Papilloma virus infection; Varicella zoster virus reactivation
P2	M	51	165	60	389	174	78	Pulmonary aspergillosis; pneumopathy
P3	F	62	50	191	140	16	30	Varicella zoster virus reactivation; Tuberculosis; Human Papilloma virus infection
P4	F	40	108	41	720	170	111	Human Papilloma virus infection
P5	M	70	42	30	330	66	165	Human Papilloma virus infection
P6	F	48	79	20	320	19	15	none
P7	M	63	227	15	440	217	53	none
P8	M	45	50	16	1300	32	1134	Pneumocystis jiroveci pneumonia
P9	M	58	4	106	500	30	15	Pneumocystis jiroveci pneumonia; Nocardia brasiliensis infection; disseminated Mycobacterium avium; recurrent Alternaria sp.; disseminated Mycobacterium kansasii
P10	F	43	220	10	820	266	208	recurrent pneumopathy
P11	F	51	200	60	300	192	93	Human Papilloma virus infection; pulmonary aspergillosis
P12	F	18	161	20	500	140	185	Campylobacter jejuni infection; Recurrent viral pulmonary infections
P13	F	43	294	3	870	294	103	none
P14	F	49	224	2	470	271	48	Past history of tuberculosis; Pansinusitis
P15	F	36	142	1	700	142	126	none
Median/Frequency	66% F	48.0	142	20.3	500	142	103	73% symptomatic
Range		18–70	4–294	1–191	140 –1300	16–294	15–1134	

### Ethics statement

Fifteen adult ICL patients were recruited into the study, which was approved by the Comité de Protection des Personnes de l'Ile de France VI. All participants gave written informed consent prior to blood sampling. Patient P9 had been previously treated with IL-2 and did not respond to therapy. CXCR4 responses for this patient have been previously reported (former code name: P3) [20]. The control group (HD, for healthy donors) consisted in 15 anonymous healthy individuals who donated blood at the Etablissement Français du Sang (Paris, France).

### Whole blood cytokine stimulation

A whole blood assay was developed to measure STAT5 phosphorylation in limiting samples from ICL patients with severe lymphocytopenia. The assay was adapted from our previously described protocol optimized for the measurement of IL-7 responses in PBMC from HIV-infected patients [36]. Briefly, 800 µl aliquots of heparinized blood were stimulated with 20 pM or 2 nM recombinant human glycosylated IL-7 (Cytheris, Issy-les-Moulineaux, France), or with 20 pM or 2 nM recombinant human IL-2 (Chiron, Emeryville, CA), or with 10 nM recombinant human TSLP (Peprotech, Rocky Hill, NJ). Blood was stimulated for 15 min at 37°C, before fixation and red cell lysis with the Lyse/Fix Phosflow buffer (BD Biosciences, San Jose, CA) for 10 min at 37°C, according to the manufacturer's instructions. After centrifugation, cells were washed with cold PBS and permeabilized by adding 90% ice-cold methanol drop by drop. After 15 min incubation on ice, methanol-fixed cells were stored at −20°C until processing for intracellular labeling.

### Intracellular phospho-specific labeling

Methanol-permeabilized cells were washed twice in ice cold PBS and rehydrated in staining buffer (PBS, 4% FBS) for 45 min. Cells were then simultaneously stained with antibodies to cell surface markers (CD3, CD4, CD45RA, and CD25) and two intracellular markers: the form of STAT5 phosphorylated at Y694 (pSTAT5) and the Treg specific factor Foxp3. The panel of antibodies was validated to ensure that recognized epitopes resisted methanol treatment and allowed the efficient measurement of pSTAT5 within the Treg and conventional CD4^+^ T cell subpopulations. Cells were stained with the following antibody combination: CD4-PerCP, CD45RA-phycoerythrin-cyanin 7 (CD45RA-PECy7) and pSTAT5-Alexa Fluor 647 (pSTAT5-AF647) from BD Biosciences; Foxp3-AF488 and CD3-allophycocyanin-e Fluor 780 (CD3-APC-eF780) from eBioscience (San Diego, CA); and CD25-PE from DAKO (Glostrup, Denmark). Each experiment included an isotypic control where the pSTAT5-specific antibody was replaced by an IgG1-AF647 antibody (BD Biosciences). After staining for 45 min at 4°C, cells were washed once in cold PBS and fluorescence was acquired on the same day on a Facs Canto flow cytometer (BD, Franklin Lakes, NJ) using the FacsDiva V software. Flow cytometry data were analyzed with the FlowJo V8.8.6 software (Tree Star, Ashland, OR). The inducible cytokine response (Δ%pSTAT5^+^) was measured by the difference in the percentage of pSTAT5^+^ cells before and after cytokine stimulation.

### Immunophenotyping and measurement of cytokine receptor expression

Since antibodies to cytokine receptor chains IL-7Rα (CD127), IL-2Rβ (CD122), γc (CD132), and TSLPR did not give detectable binding to methanol-permeabilized cells, a separate set of experiments was carried out to evaluate expression of these markers on unpermeabilized whole blood cells. Samples of 400 µl heparinized whole blood were lysed with BD Pharm Lyse™ Buffer (BD Biosciences) for 10 min. at 37°C. After centrifugation and washing, cells were resuspended in cell surface staining buffer (PBS, 1% BSA, 10 nM sodium azide) and were labeled for 30 min at 4°C with combinations of the following antibodies: CD122-PE, CD132-PE, HLA-DR-PE, CD3-PerCP, CD4-PerCP and CD45RA-PECy7 from BD Biosciences; CD4-FITC, CD25-APC, CD127-APC-AF750, CD62L-APC-AF750 and CD3-APC-eF780 from eBioscience; and TSLPR-PE from BioLegend (San Diego, CA). Cells were either fixed directly in 1% paraformaldehyde, or permeabilized with a Fix/Perm buffer adapted to Foxp3 detection (eBioscience), labeled for 30 min with the Foxp3-AF488 antibody, washed and fixed in paraformaldehyde. Fluorescence was acquired on a Facs Canto cytometer as described above.

### Measurement of cytokines in plasma

IL-7 concentration was measured in plasma collected on EDTA, using an ELISA immunoassay according to the manufacturer's instructions (Quantikine HS human IL-7, R&D Systems). IL-2 concentration was measured in plasma collected on EDTA, using a bead-based immunoassay (Milliplex, from Merck Millipore, Billerica, MA), according to the manufacturer's instructions.

### Statistical analyses

Analyses were performed with the Prism 5.0 software (GraphPad Software, La Jolla, CA), using nonparametric statistical tests in all cases. Differences in variables between groups were analyzed with the Mann-Whitney U Test. Horizontal bars on data plots indicate median values. Correlations were analyzed with Spearman's coefficient R. All significant differences between groups (p<0.05) are reported on data plots.

## Results

### 1 Increased immune activation in ICL

Eleven of the 15 ICL patients studied experienced at least one infectious episode (Table 1). The spectrum of opportunistic pathogens included fungi (*Pneumocystis jiroveci, Aspergillus niger*), viruses (human papilloma virus, varicella zoster virus) and bacteria (*Nocardia brasiliensis*), with a predominance of mycobacteria (*M. tuberculosis, M. avium, M. kansasii*). The patients had a median age of 48 years [range: 18–70] and had been diagnosed with ICL for a median duration of 20 months [1–191]. The degree of CD4^+^ T cell depletion was variable (median CD4^+^ T cell count: 142/mm^3^ [16–294]) and did not strictly correlate with the severity of symptoms. However, ICL patients with severe opportunistic infections had CD4^+^ T cell counts <200/mm^3^. Most ICL patients (14/15) also had CD8^+^ T cell depletion, as indicated by a median CD8^+^ T cell count of 103/mm^3^ [15–1134] and by a relatively preserved CD4/CD8 ratio (median ratio: 1.28 [0.03–5.6]).

Whole blood phenotyping analyses showed a decrease in the percentage of CD45RA^+^ CD4^+^ T cell population in ICL patients as compared to healthy blood donors (Table 2; p = 0.0005). Further analyses of T CD4^+^ subsets based on combination of the CD45RA and CD62L markers confirmed a marked decrease of the naive subset (CD45RA^+^ CD62L^+^; p<0.0005) and showed a converse increase in the effector memory subset (CD45RA^−^ CD62L^−^; p<0.0005), while the central memory-like subset (CD45RA^−^ CD62L^+^) showed no significant changes. Expression of the activation marker HLA-DR was significantly increased in CD4^+^ T cells of ICL patients (p<0.01; Table 2). A concomitant decrease in expression of the alpha chain of the IL-7 receptor, CD127, occurred in the same population (p<0.05). HLA-DR and CD127 expression correlated negatively (R_ICL_ = −0.51, p<0.05), consistent with the decrease in CD127 being driven by immune activation in ICL. Expression of the different chains of the IL-2 receptor, the alpha chain CD25, the beta chain CD122, and the common γc chain CD132, did not show significant changes in the ICL group. The last cytokine receptor chain analyzed, TSLPR, is a γc-like protein that can associate with CD127 to form the TSLP receptor. TSLPR is expressed at very low levels on human T cells but can be induced upon TCR activation [37]. Interestingly, TSLPR expression could be detected in memory CD4+ T cells from ICL patients, as shown in a representative example (Fig. S1, row C). The increase in TSLPR expression was highly significant in the ICL group (p<0.005), even though the frequency of TSLPR+ cells remained relatively low (median value: 2.7%; range: 1.2%–10.3%). Taken together, immunophenotyping of the CD4^+^ T cell population pointed to a series of changes consistent with chronic immune activation in ICL, including a switch toward more differentiated memory subsets, the induction of activation markers (HLA-DR, TSLPR), and the downregulation of CD127.

**Table 2:** Immunophenotype of the CD4+ T cell population.

	HD group (n = 15)	ICL group (n = 15)	P value
% CD4+ in T CD3+	68.3 (43.9–77.5)	47.1 (2.4–83.0)	**0.03**
**In T CD4+:**			
% CD45RA+	57.02 (28.6–71.1)	24.4 (1.6–65.9)	**0.0005**
% CD45RA+ CD62L+	55.2 (29.4–68.8)	21.0 (0.7–54.2)	**0.0002**
% CD45RA+ CD62L−	1.4 (0.2–4.9)	2.5 (0.2–11.7)	0.17
% CD45RA− CD62L+	31.7 (18.4–50.1)	36.0 (6.2–54.5)	0.50
% CD45RA− CD62L−	11.9 (9.2–30.8)	39.8 (21.9–85.8)	**0.0002**
% HLA-DR	1.3 (0.34–3.6)	3.7 (0.5–7.3)	**0.006**
% CD127	78.3 (58.0–89.4)	72.4 (32.8–89.6)	**0.027**
% CD25	8.4 (4.6–12.7)	11.6 (5.6–43.7)	0.053
% Treg (Foxp3+ CD25hi)	4.1 (1.1–8.3)	6.0 (1.2–20.40)	0.051
CD132 MFI	220 (148–927)	261 (93–404)	0.61
% CD122	3.7 (1.1–14.8)	3.0 (1.52–16.6)	0.90
% TSLPR	1.1 (0.8–3.0)	2.8 (1.8–10.6)	**0.0017**
**In T CD4+ RA−:**			
% HLA-DR	2.1 (0.6–5.3)	5.4 (0.6–8.5)	**0.027**
% CD127	77.8 (59.0–92.8)	72.6 (48.0–89.2)	0.092
% CD25	14.2 (4.9–20.9)	14.5 (6.4–43.7)	0.90
% Treg (Foxp3+ CD25hi)	6.4 (1.6–9.1)	6.0 (2.0–21.9)	0.60
CD132 MFI	225 (158–1054)	253 (91–399)	0.92
% CD122	5.0 (1.2–22.1)	3.6 (1.7–19.2)	0.47
% TSLPR	1.0 (0.6–2.3)	2.7 (1.2–10.3)	**0.0009**

The median and range are reported for each immunological parameter. P values obtained by comparing the HD and ICL groups with the Mann-Whitney U test are reported. HD: healthy donors; ICL: patients with Idiopathic CD4 Lymphocytopenia; MFI: mean fluorescence intensity.

It remained possible that increases in immune activation markers resulted from changes in proportions of CD4^+^ T cell subsets, considering that such markers have a physiologically higher expression in the memory than in the naive compartment. Therefore, we further compared activation marker expression within the T CD4^+^ CD45RA^−^ memory compartment. HLA-DR and TSLPR expression remained significantly increased in the ICL group (p<0.05 and p<0.0005, respectively; Table 2) while the decrease in CD127 expression lost significance (p = 0.09). Thus, immunophenotype changes in ICL patient CD4^+^ T cells resulted from both changes in differentiation patterns and in immune activation status. Since the naive CD4^+^ T cell compartment was virtually absent in a subgroup of ICL patients (minimum: 0.7%), further analyses of cytokine responses focused predominantly on the T CD4^+^ CD45RA^−^ memory population.

### 2 Decreased responses to IL-7 in ICL

**Figure 1:**
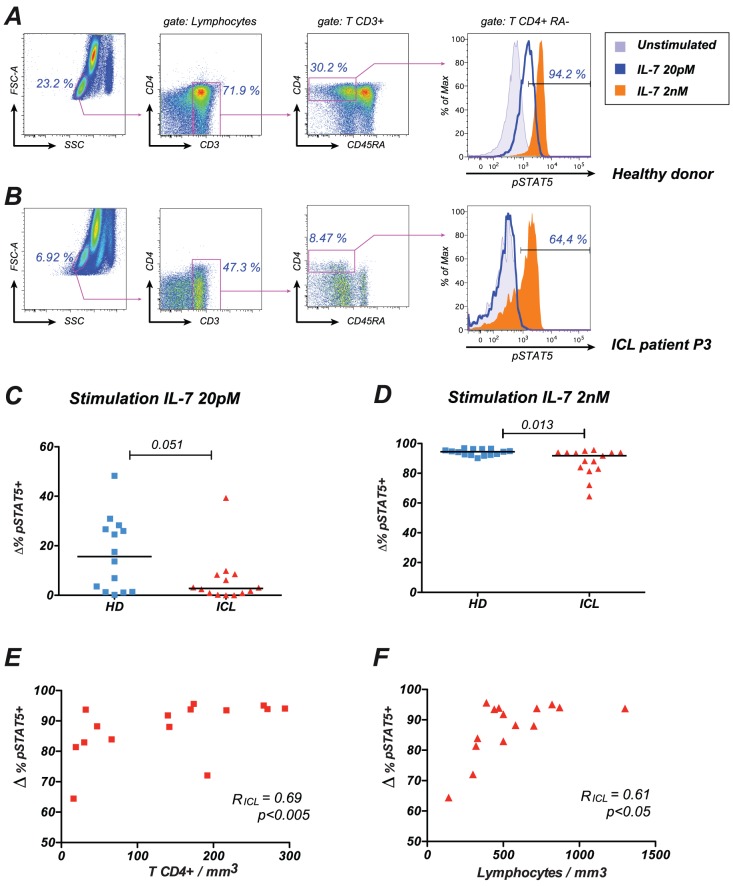
Decreased IL-7 responses in ICL. (A) Example of pSTAT5 response to IL-7 stimulation measured in whole blood from a healthy donor. The gating strategy to detect pSTAT5 induction by flow cytometry within the memory CD4^+^ T cell population, defined as CD3^+^ CD4^+^ CD45RA^−^ lymphocytes, is depicted. The percentages within the histogram plots indicate the fraction of pSTAT5^+^ cells after high dose IL-7 stimulation. A dose dependent response is observed, with a higher pSTAT5 expression at high IL-7 dose (2 nM) than at low IL-7 dose (20 pM). (B) Example of decreased pSTAT5 response to IL-7 stimulation in memory CD4^+^ T cells from ICL patient P3. (C) Induction of pSTAT5 after stimulation with low dose IL-7. The inducible cytokine response (Δ%pSTAT5^+^) was measured by the difference in the percentage of pSTAT5^+^ cells before and after IL-7 stimulation in memory CD4^+^ T cells. (D) Induction of pSTAT5 after stimulation with high dose IL-7. HD: healthy donors; ICL: patients with idiopathic CD4 lymphocytopenia. P values obtained with the Mann-Whitney U test are reported. (E) Correlation between pSTAT5 responses to high dose IL-7 stimulation and absolute CD4^+^ T cells counts in the blood of ICL patients. (F) Correlation between pSTAT5 responses to high dose IL-7 stimulation and the total lymphocyte count in the blood of ICL patients. The Spearman correlation coefficient R and the corresponding P value are indicated.

Responses to IL-7 stimulation were evaluated by measuring the degree of phosphorylation of STAT5 at tyrosine 694 by intracellular labeling. Whole blood samples were stimulated with either a low IL-7 dose (20 pM) close to the Kd of the receptor [38], [39] or with a high IL-7 dose (2 nM) that ensured full occupancy of the receptor. Detection of phosphorylated STAT5 (pSTAT5) showed a dose-dependent response within the T CD4^+^ CD45RA^−^ memory population (Fig. 1A). Analysis of basal pSTAT5 levels prior to stimulation showed low levels of constitutive STAT5 activation that did not differ significantly between groups (median percentage of pSTAT5+ cells: 0.65% in HD; 0.89% in ICL; p≥0.05; not shown). Low dose IL-7 stimulation induced variable responses in blood from healthy donors, the percentage of pSTAT5^+^ memory CD4^+^ T cells ranging from 0 to 48% (Fig. 1C). Responses in the ICL group tended to be in the lower range (HD vs. ICL: p = 0.051). High dose IL-7 stimulation induced a uniformly high percentage of response in healthy donors (range: 90.1–96.8% pSTAT5+ cells), consistent with a full occupancy of the receptor (Fig. 1D). Interestingly, high dose IL-7 responses were significantly lower in the ICL group (range: 64.4–95.6%; p = 0.01), with a subset of ICL patients showing clear impairment of STAT5 activation (example in Fig. 1B). pSTAT5 responses to high dose IL-7 correlated with absolute CD4^+^ T cell counts in ICL patients (Fig. 1E, R_ICL_ = 0.69, p<0.005), suggesting that impaired IL-7 responses may play a role in the CD4^+^ T cell loss characteristic of ICL. In addition, low IL-7 responses were associated with a low total lymphocytes count (Fig. 1F, R_ICL_ = 0.61, p<0.05), pointing to a general perturbation of leukocyte homeostasis in ICL.

**Figure 2:**
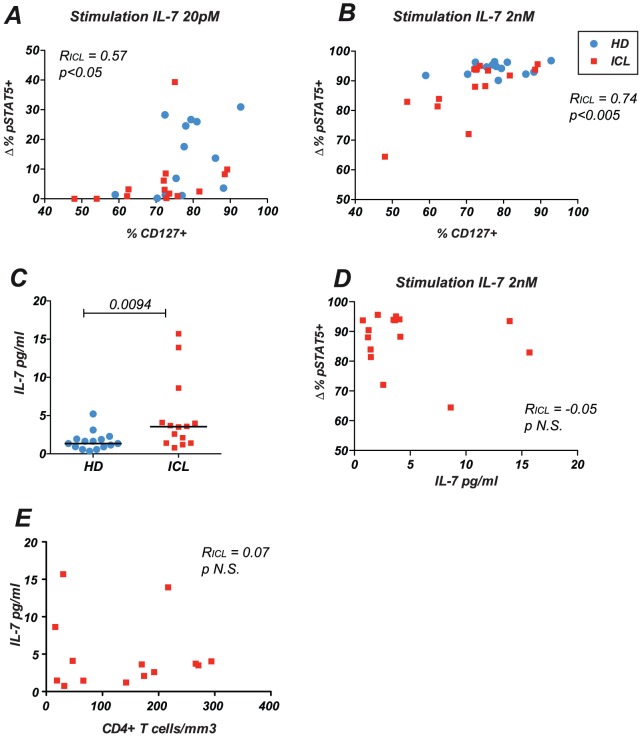
Correlation between IL-7 responses and CD127 expression. (A and B) The percentage of IL-7-induced pSTAT5^+^ cells within the memory CD4^+^ T cell population is reported as a function of CD127 expression. (A) Responses to low dose IL-7 (B) Responses to high dose IL-7. HD: healthy donors; ICL: patients with idiopathic CD4 lymphocytopenia. The Spearman rank correlation coefficient R_ICL_ computed for ICL patients is reported. (C) Concentration of IL-7 in plasma measured by ELISA assay. (D) Lack of correlation between plasma IL-7 and pSTAT5 responses to high dose IL-7 in memory CD4^+^ T cells of ICL patients. (E) Lack of correlation between plasma IL-7 and CD4^+^ T cell counts in ICL patients.

Analysis of pSTAT5 responses in function of CD127 expression showed a positive correlation between the two parameters in the ICL group (Fig. 2A and 2B). The correlation was significant after both high dose IL-7 stimulation (R_ICL_ = 0.74; p<0.005) and low dose IL-7 stimulation (R_ICL_ = 0.57; p<0.05), suggesting the physiological relevance of this finding over a wide range of IL-7 concentrations. These findings implicated the downregulation of the IL-7 receptor as a probable cause for the low IL-7 responses in ICL.

CD127 can be downregulated by its ligand, the IL-7 cytokine [40], and circulating IL-7 concentration is known to increase in situations of lymphopenia [22], [23], raising the possibility that chronic IL-7 stimulation in ICL may drive CD127 downregulation and cause a desensitization of IL-7 responses. To explore this notion, we measured IL-7 concentrations in the plasma of ICL patients (Fig. 2C). Circulating IL-7 was indeed increased in ICL patients as compared to healthy donors (p = 0.009), though we noted that IL-7 levels showed a wide distribution in patients. Circulating IL-7 did not correlate with the decrease in IL-7 responses measured in memory CD4^+^ T cells (Fig. 2D). The concentration of IL-7 did not correlate either with CD127 expression (not shown) or with CD4^+^ T cell counts (Fig. 2E). Thus, the increase in circulating IL-7 did not appear sufficient to account for defective IL-7 responses in ICL.

IL-7-dependent signaling was also evaluated in the naive CD45RA^+^ CD4^+^ T cell compartment, this analysis being limited to patients for whom it was possible to collect a sufficient number of events in the naive gate (ICL: n = 11). Responses to low dose IL-7 (Fig. S2A) and high dose IL-7 (Fig. S2B) tended to be lower in the ICL group than in healthy donors, though the differences did not reach significance (p = 0.11 for low dose IL-7; p = 0.13 for high dose IL-7). It should be considered, however, that patients with a minimal naive CD4^+^ T cell population, and presumably a more severe perturbation of CD4^+^ T cell homeostasis, had to be excluded from this analysis. The correlations between IL-7 responses and CD127 expression in the naive CD45RA^+^ CD4^+^ T cell population reached significance at high dose but not at low dose IL-7 stimulation (Fig. S2C and S2D). The responses of naive CD4^+^ T cells to high dose IL-7 correlated with absolute CD4^+^ T cell counts in ICL patients (Fig. S2E, R_ICL_ = 0.65, p<0.05), emphasizing the relevance of impaired IL-7 responses in the loss of CD4+ T cell homeostasis characteristic of ICL.

### 3 Decreased responses to IL-2 in ICL

**Figure 3:**
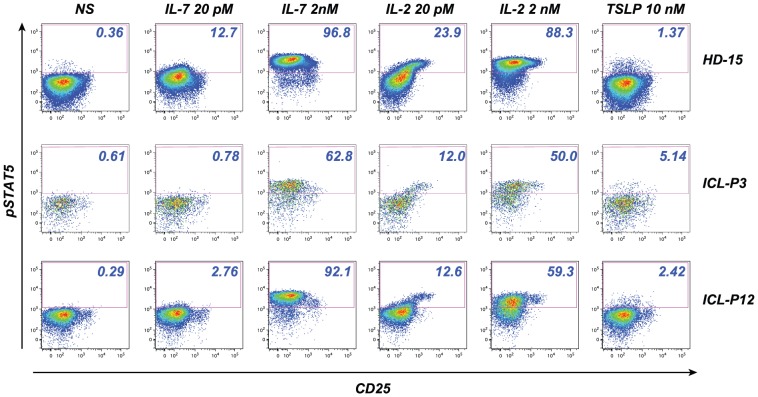
Cytokine responses in function of CD25 expression. Examples of pSTAT5 responses to IL-7, IL-2, and TSLP stimulation are shown in the CD4+ CD45RA- T cell population for one healthy donor (HD-15) and two ICL patients (ICL-P3 and ICL-P12). The percentage of pSTAT5+ cells is reported in the upper right corner of each dot plot. Plots in the first column represent non-stimulated cells (NS). Responses to low dose IL-2 were highly dependent on the degree of CD25 expression (4th column). In contrast, IL-7 responses tended to be lower in the CD25hi population (2nd and 3rd columns), consistent with known properties of Tregs [36]. TSLP responses did not show a dependence on CD25 expression (6th column).

**Figure 4:**
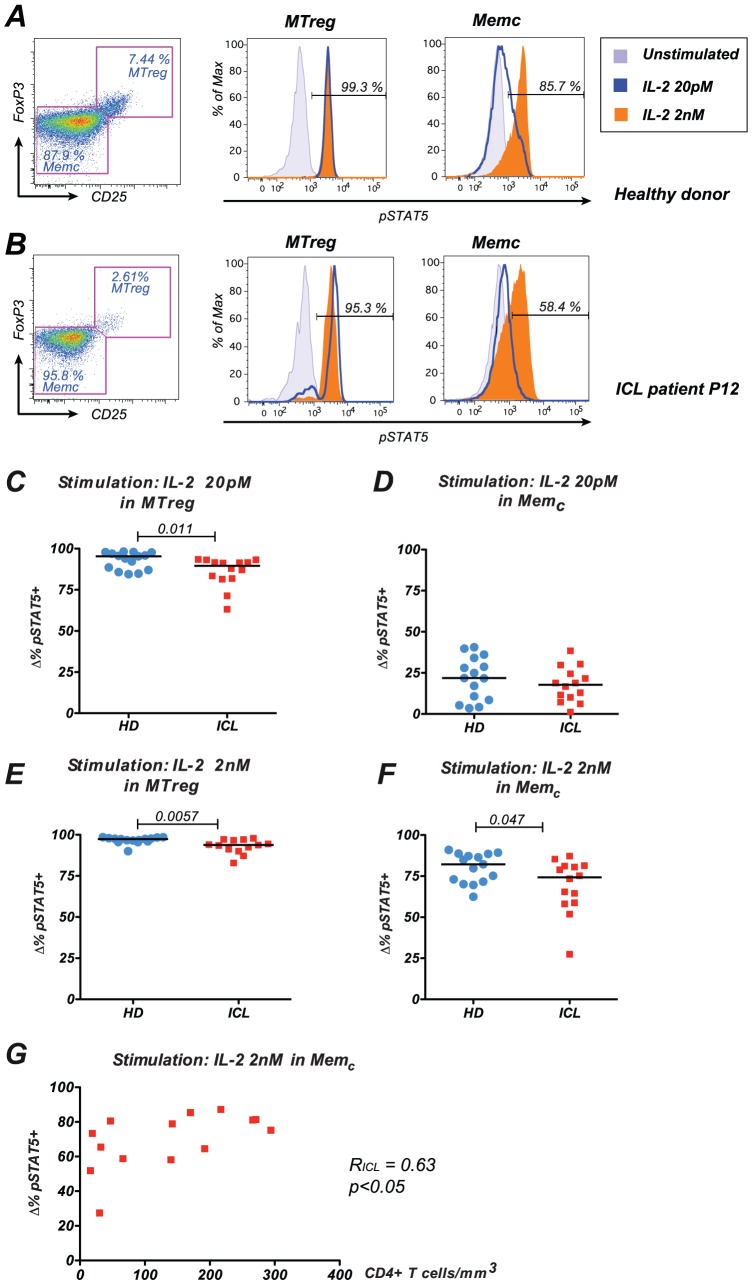
Decreased IL-2 responses in ICL. (A) Example of pSTAT5 response to IL-2 stimulation measured in whole blood from a healthy donor. The Foxp3/CD25 gating used to defined memory Tregs (MTreg) and conventional memory CD4^+^ T cells (Memc) within the CD3^+^ CD4^+^ CD45RA^−^ population is reported (left plot). Histograms show pSTAT5 basal levels (light blue area) and pSTAT5 induction after stimulation with low dose IL-2 (blue line) or high dose IL-2 (orange area) within the MTreg (middle plot) and Memc (right plot) populations. The percentages within each plot indicate the fraction of pSTAT5^+^ cells after high dose IL-2 stimulation. Maximal response is already achieved at low IL-2 concentration in MTregs, while a dose-dependent response is observed in Memc. (B) Example, of IL-2 responses in CD4^+^ T cells from an ICL patient. A small fraction of MTregs fail to respond to IL-2 (middle plot), while a larger fraction of Memc cells appear unresponsive (right plot). (C to F) The inducible cytokine response (Δ%pSTAT5^+^) was measured by the difference in the percentage of pSTAT5^+^ cells before and after IL-2 stimulation. HD: healthy donors; ICL: patients with idiopathic CD4 lymphocytopenia. P values obtained with the Mann-Whitney U test are reported. (C) Low dose IL-2 response in MTreg; (D) Low dose IL-2 response in Memc; (E) High dose IL-2 response in MTreg; (F) High dose IL-2 response in Memc. (G) Correlation between pSTAT5 responses of Memc to high dose IL-2 stimulation and absolute CD4^+^ T cells counts in the blood of ICL patients. The Spearman correlation coefficient R and the corresponding p value are indicated.

An initial analysis of pSTAT5 induction in function of CD25 expression showed that IL-2 responses were highly dependent on CD25 levels (see examples for one healthy donor and two ICL patients in Fig. 3). Since regulatory T cells (Tregs) express significantly more CD25 than conventional memory CD4^+^ T cells (Memc), IL-2 responses were analyzed separately within the two populations. The memory Treg subset (MTreg) was defined according to Foxp3 and CD25 expression within the T CD4^+^ CD45RA- population (Fig. 4A). In a representative healthy donor, MTregs responded as efficiently to low dose (20 pM) and high dose (2 nM) IL-2 stimulation as measured by pSTAT5 induction, consistent with a high expression of the high affinity IL-2 receptor in this subset (Fig. 4A, middle panel). In contrast, the Memc subset showed only partial response to low dose IL-2, while most Memc cells responded to high dose IL-2 (Fig. 4A, right panel). Interestingly, a small subset of MTregs appeared unresponsive to IL-2 in ICL patient P12 (Fig. 4B, middle panel), while IL-2 responses appeared also decreased in the Memc population (Fig. 4B, right panel).

When considering all study subjects, in vitro stimulation with low dose IL-2 (20 pM) induced a high response rate in MTregs in the HD group (median: 95.3% pSTAT5^+^ cells) that was significantly decreased in the ICL group (median: 89.6% pSTAT5^+^ cells, p<0.05) (Fig. 4C). Low dose IL-2 stimulation led to a moderate response rate within the Memc subset, that was comparable between the HD and ICL groups (p N.S., Fig. 4D). Analysis of responses to high dose IL-2 (2 nM) confirmed the defect within the MTreg subset of ICL patients (p<0.01, Fig. 4E) and revealed an additional defect in the Memc subset (p<0.05, Fig. 3F). Importantly, decreased IL-2 responses in Memc correlated with decreased CD4^+^ T cell counts in the blood of ICL patients (R_ICL_ = 0.63, p<0.05, Fig. 4G), suggesting that defective IL-2 responses may contribute to the loss of CD4^+^ T cell homeostasis.

IL-2 responses were measured in the naive CD45RA^+^ CD4^+^ T cell population for patients who had sufficient cells available for analysis (ICL: n = 10). The induction of pSTAT5 after 20 pM IL-2 stimulation was low in the naive subset, as expected, and did not differ significantly between groups (median Δ%pSTAT5+ cells: 2.9% in HD; 7.5% in ICL; p≥0.05; not shown). High dose IL-2 responses in the naive subset did not differ either (median Δ%pSTAT5+ cells: 34.6% in HD; 44.2% in ICL; p≥0.05; not shown). Thus, the impairment of IL-2 responses appeared restricted to the memory CD4^+^ T cell population.

**Figure 5:**
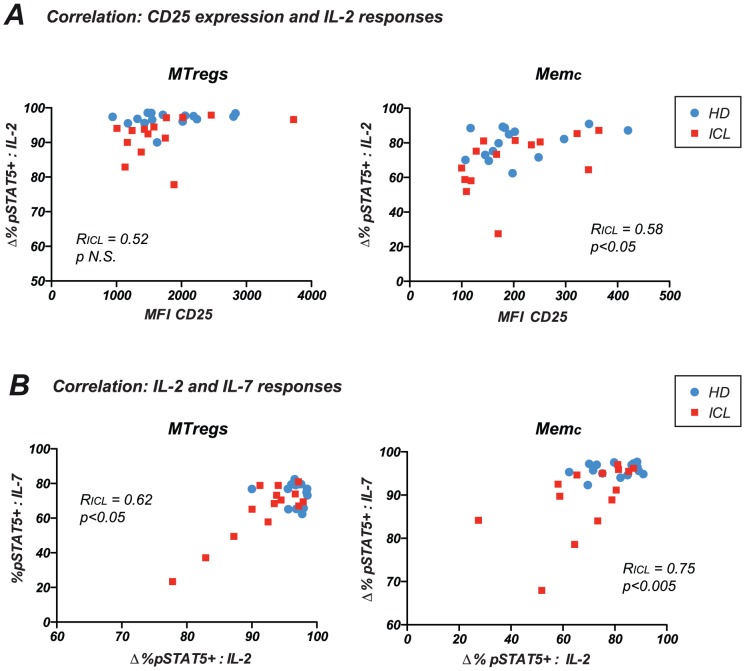
Impairment of IL-2 responses correlate with lower CD25 expression and decreased IL-7 responses. (A) The percentage pSTAT5^+^ cells induced after high dose IL-2 stimulation (2 nM) is reported as a function of CD25 expression in MTregs (left plot) and Memc (right plot) populations. The mean fluorescence intensity (MIF) of CD25 is reported on the x axis. (B) Correlation between pSTAT5 responses to high dose IL-2 and high dose IL-7 in MTregs (left plot) and Memc (right plot). HD: healthy donors; ICL: patients with idiopathic CD4 lymphocytopenia. The Spearman rank correlation coefficient R_ICL_ computed for ICL patients is reported.

Correlation analysis between high dose IL-2 responses and CD25 expression showed a positive association between the two parameters in the Memc population (Fig. 5A, right). The correlation was significant for ICL patients (R_ICL_ =  0.58; p<0.05), suggesting that a relative decrease in CD25 expression could impact IL-2 responses in ICL. Correlations were not significant in the HD group, but reached statistical significance when analyzing all study subjects together (R_ALL_ = 0.51 and p<0.001 in Memc). In contrast, the lack of correlation between CD25 expression and IL-2 responses in MTregs suggested that CD25 expression was not a limiting factor in this subset, possibly due to a constitutively high expression. Observing the distribution of data points in Fig. 5A (left panel) suggested that, for comparable CD25 expression, MTregs of ICL patients had lower IL-2 responses than MTregs of healthy controls. Thus, defective IL-2 responses in ICL could not be solely explained by a decreased expression of CD25. To determine whether a high level of circulating IL-2 may lead to a desensitization of IL-2 responses, we measured IL-2 concentrations in the plasma of healthy donors and of a subset of ICL patients (n = 7). We did not detect increased IL-2 levels in the tested ICL patients (median values: 20.6 pg/ml in HD; 11.8 pg/ml in ICL; P≥0.05; not shown), suggesting that an excess of IL-2 was unlikely to account for the impairment of IL-2 responses.

Interestingly, a positive correlation was detected between IL-2 and IL-7 responses (Fig. 5B), suggesting that common mechanisms may perturb several cytokine systems in ICL. High dose IL-7 and IL-2 responses correlated positively for ICL patients, both in MTregs (R_ICL_ = 0.62; p<0.05) and in the Memc subset (R_ICL_ = 0.74; p<0.005). Taken together, these findings showed that ICL patients with impaired IL-2 responses tended to have also weak IL-7 responses, which, combined together, may significantly perturb CD4^+^ T cell homeostasis.

### 4 Compensatory increase in TSLP responses in ICL

**Figure 6:**
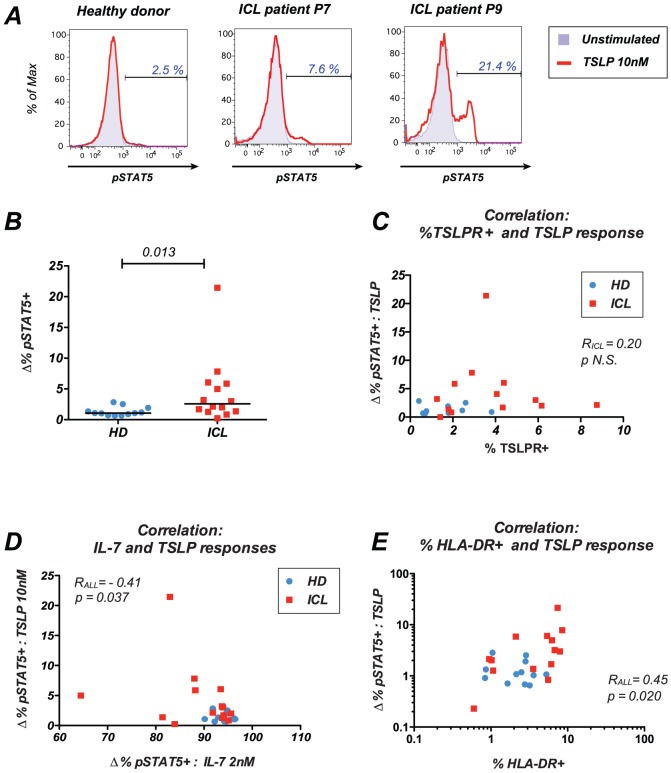
Induction of a compensatory TSLP response in ICL. (A) Examples of pSTAT5 responses to TSLP stimulation in one representative healthy donor (left plot) and ICL patients P7 and P9 (middle and right plots). Whole blood was stimulated with TSLP 10 nM and evaluated for the expression of phosphorylated STAT5 (pSTAT5) in memory CD4+ T cells. (B) pSTAT5 response after stimulation with high dose TSLP within the memory CD4^+^ T cell population. The inducible cytokine response (Δ%pSTAT5^+^) was measured by the difference in the percentage of pSTAT5^+^ cells before and after stimulation with 10 nM TSLP. (C) Lack of correlation between expression of the TSLPR receptor chain and TSLP responses. The inducible TSLP response (Δ%pSTAT5^+^) cells within the CD3^+^ CD4^+^ CD45RA^−^ population is reported in function of TSLPR expression. Blue circles: healthy donors; red squares: ICL patients. (D) Inverse correlation between IL-7 and TSLP responses. The inducible pSTAT5 responses to high dose IL-7 (x axis) and high dose TSLP (y axis) with memory CD4^+^ T cells are plotted. (E) Positive correlation between HLA-DR expression and TSLP responses. The percentage of HLA-DR^+^ cells (x axis) and the inducible TSLP response (y axis) within the memory CD4^+^ T cell population are plotted. HD: healthy donors; ICL: patients with idiopathic CD4 lymphocytopenia. The Spearman rank correlation coefficient R_ALL_ computed for all study subjects is reported.

We next evaluated STAT5 phosphorylation in response to high dose TSLP stimulation (10 nM) in whole blood (examples in Fig. 6A and Fig. 3, right column). The percentage of responder CD45RA^−^ CD4^+^ T cells was low in the HD group (median pSTAT5^+^ cells: 1.1% [0.6–2.8]), consistent with the known low reactivity of human T cells to this cytokine [37], [41] (Fig. 5B). Intriguingly, TSLP responses were increased in memory CD4^+^ T cells of ICL patients (2.57% [0.2–21.4]; p = 0.013). The increase remained significant when the patient with a particularly high TSLP response (patient P9: 21.4% pSTAT5+ cells) was excluded from the analysis (p = 0.02), indicating that acquired TSLP responsiveness was a frequent occurrence in ICL, even though the percentage of responding cells remained generally low. TSLP responses remained minimal in the naive CD45RA^+^ CD4^+^ T cell compartment (not shown), suggesting that only memory cells could become responsive to this cytokine.

Since CD127 expression was decreased rather than increased in ICL, it was unlikely that this receptor chain accounted for the changes in TSLP response. Indeed, CD127 expression did not correlate with TSLP responses (p = 0.37). The other chain of the receptor, TSLPR, showed a significantly higher expression in the ICL group (Table 2, p<0.001), suggesting that it played a role in the increased sensitivity of memory CD4^+^ T cells to TSLP. However, there was no positive correlation between the two parameters (Fig. 6C, p = 0.52), indicating that other parameters than receptor expression influenced the reactivity of CD4^+^ T cells to TSLP. Comparison of pSTAT5 responses to different cytokines revealed an inverse correlation between TSLP and IL-7 responses, which did not reach statistical significance in the ICL group alone (p = 0.23) but was significant when considering all study subjects (Fig. 6D, R_ALL_ = −0.41, p<0.05). Given the shared receptor chain and the similar functions of the two cytokines, this finding pointed to a possible role for TSLP in compensating decreased IL-7 responses and maintaining minimal CD4^+^ T cell homeostasis. Further analyses showed that TSLP responses correlated positively with HLA-DR expression (Fig. 6E), both in ICL patients (R_ICL_ = 0.59, p<0.05) and when considering all study subjects together (R_ALL_ = 0.45, p<0.05). Thus, immune activation appeared to promote the unusual sensitivity of CD4^+^ T cells to TSLP. Taken together, these findings suggested the induction of a TSLP-based homeostatic mechanism in situations of abnormal immune activation and defective γc cytokine responses.

## Discussion

This study provides evidence for altered cytokine responses in ICL. The two major γc family cytokines involved in the control of CD4^+^ T cell homeostasis, IL-7 and IL-2, induced suboptimal STAT5 phosphorylation in memory CD4^+^ T cells of ICL patients, which correlated with decreased CD4^+^ T cell counts. The defects in IL-7 and IL-2 responses were partial but correlated together, resulting in a combined defect that may significantly impact CD4^+^ T cell survival and renewal capacities, and thus may contribute to ICL pathogenesis. Abrogation of IL-7-dependent signaling results in a SCID phenotype characterized by the lack of T cells and profound immunodeficiency [24], while abrogation of IL-2-dependent signaling leads to inflammation, multi-organ lymphocyte infiltrates, and autoimmunity in mice [33] and possibly in humans [42]. Both immune deficiencies and autoimmune manifestations have been reported in ICL [12], which appears consistent with an impairment of both IL-7 and IL-2 systems. It is relevant that Tregs showed decreased IL-2 responses in ICL, which may limit their persistence and suppressive function [43] and contribute to the abnormal immune activation characteristic of the disease.

Importantly, ICL was not characterized by a general impairment of STAT5 phosphorylation, as indicated by increased responses to the cytokine TSLP. Rather, proximal defects in the IL-2 and IL-7 signaling pathways likely account for the decreased responses to these cytokines. We did not find evidence for altered expression of the common receptor chain γc in CD4^+^ T cells of ICL patients. In contrast, expression of the alpha chain of IL-7R, CD127, was downregulated, and clearly correlated with decreased STAT5 phosphorylation after IL-7 stimulation (R = 0.74). Chronic immune activation is known to induce the internalization of CD127 and its transcriptional repression [40], [44], and thus may underlie the defect in IL-7 responses in ICL. A similar phenomenon occurs in HIV infection, where abnormal immune activation leads to chronically low CD127 expression in memory T cells and contributes to impaired IL-7 responses [36], [45]. Of note, a recently published study also reports an impairment of IL-7 and IL-2 responses in ICL patients [46], supporting the notion that defective cytokine responses contribute to the loss of CD4^+^ T cell homeostasis in ICL. In this study, decreased IL-7 signaling did not correlate with CD127 downregulation in CD4^+^ T cells, even though CD127 expression was decreased as compared to healthy controls. Thus, additional factors may impair IL-7 signaling independently of IL-7 receptor expression. One possibility may be a desensitization of the STAT5 signaling pathway due to chronic stimulation by high IL-7 concentrations, as suggested by Puronen et al. [46]. However, the levels of IL-7 in plasma did not correlate with the decrease of pSTAT5 responses in our ICL cohort, suggesting that the increase in circulating IL-7 is not sufficient either to account for the impairment of IL-7 signaling. Thus, multiple factors, including chronic immune activation and increases in circulating cytokines levels, may concur in impairing the responsiveness of the STAT5 pathway. It was intriguing that some ICL patients with very low CD4^+^ T cell counts did not show an increase in circulating IL-7 (Fig. 2E), as would have been predicted by the ‘IL-7 consumption’ model. The lack of IL-7 increase in a subset of ICL patients, which has also been reported in the NIH ICL cohort [23], raises the possibility of a defective production of this key homeostatic cytokine. It should be noted that the levels of IL-7 measured in the circulation may not necessarily reflect those found within lymphoid tissues, as suggested by a recent study showing reduced IL-7 availability in lymph nodes of patients with advanced HIV infection [47]. Thus, it will be important in future studies to directly evaluate the production of IL-7 by stromal cells within lymphoid organs of ICL patients.

ICL patients showed an impairment of IL-2 responses that affected both conventional memory CD4^+^ T cells and the Treg population. The decrease in IL-2 signaling is of lower magnitude than that seen for IL-7, but it could still be physiologically relevant through its effect on Tregs, which are highly dependent on IL-2 for their homeostatic regulation. The mechanism for the impairment of IL-2 responses remains to be elucidated, though a low expression of CD25 may be a contributing factor in a subset of ICL patients, given the correlation between these two parameters in the Memc population. CD25 is considered as an activation marker in conventional memory cells [48] and it is therefore intriguing that CD25 expression is not increased in ICL patients, in spite of ongoing chronic immune activation. Exploration of the multiple transcriptional and post-transcriptional mechanisms that regulate CD25 expression [49] may further inform on ICL pathogenesis. CD25 expression may not be the only factor involved in the impairment of IL-2 responses, since the two parameters did not correlate in the MTreg population. Of note, defective IL-2 responses in Tregs may have far reaching consequences, considering that inactivation of IL-2 signaling is known to inhibit Treg suppressive function and consequently to increase immune activation [43]. Activation can in turn lead to decreased CD127 expression and impaired IL-7 responses. Thus, a defect in IL-2 response can perturb IL-7 responses, which may explain the correlation between these two parameters in ICL.

The finding of perturbed IL-2 and IL-7 responses is compatible with our previous observation for decreased CXCR4 expression in ICL [20]. Cytokines of the γc family are major inducers of CXCR4 [30]–[32] and decreased IL-2 and IL-7 responses are expected to lower the expression of this chemokine receptor. As a consequence, the loss of CD4^+^ T cell homeostasis in ICL may not result only from lack of trophic and survival signals from cytokines, but also from perturbed CXCR4-dependent trafficking, which would impair T cell differentiation. Importantly, high amounts of exogenous cytokine appear to reverse the CXCR4 expression defect, as indicated by normalized CXCR4 levels at the surface of circulating CD4^+^ T cells after IL-2 immunotherapy in three out of four treated patients [20]. In this initial study, the only patient who did not normalize CXCR4 expression levels also failed to increase CD4^+^ T cell counts after IL-2 treatment and did not benefit clinically from the therapy, with continued occurrence of multiple opportunistic infections. Of note, this patient, denoted P9 in the present study, showed the most severely impaired IL-2 responses *in vitro*. Testing cytokine responses *in vitro* should be evaluated further, to determine whether this assay may indeed have predictive value and help clinicians devise immunotherapeutic approaches to treat ICL. In this respect, it is relevant that IL-7 responses appeared less defective than IL-2 responses for patient P9, raising the possibility that IL-7 therapy may benefit this type of patient. Phase I/II studies of IL-7 immunotherapy in lymphodepleted cancer patients and HIV infected patients have demonstrated the capacity of this cytokine to reconstitute both the naive and memory CD4^+^ T cell pools and, importantly, to increase TCR repertoire diversity, which should help restore adaptive responses to diverse pathogens [50], [51].

Naive CD45RA+ CD4+ T cells were severely depleted in ICL patients, consistent with previous studies [5], [12], [26]. Analyses of cytokine responses in this subset showed a trend for decreased IL-7 responses, but preserved IL-2 responses and an absence of reactivity to TSLP. Thus, the remaining naive CD4+ T cells were few but appeared to have relatively preserved functions. One straightforward explanation for the loss of naive CD4+ T cells may be their conversion to memory-like cells as a homeostatic response to severe lymphopenia, as described in mouse models of T cell depletion and in patients undergoing myeloablative therapies [33], [52]. Upon T cell depletion, increased availability of γc-cytokines, in particular IL-7 and IL-15, and of self antigens presented by dendritic cells can drive the conversion of naive cells to a CD45RA-negative phenotype and promote their proliferation, in the absence of TCR stimulation by exogenous antigens. The increase in CD4+ T cell proliferation observed in ICL patients [12] suggests that conversion of naive cells to memory-like proliferating cells is occurring in ICL, in spite of the suboptimal responses to γc-cytokines. This homeostatic proliferation could contribute to the immune activation characteristic of ICL, though other mechanisms may also be at work. Direct consequences of CD4+ T cell loss, such as the reactivation of opportunistic pathogens, or microbial translocation due to the depletion of Th17 cells in the gut mucosa, likely contribute to chronic immune activation in ICL, in a pattern comparable to that seen in HIV infection [53]. Indeed, microbial translocation products can be detected in the plasma of ICL patients [13]. The lower IL-2 responses in ICL patients may also fuel immune activation through reduced Treg suppressive activity, as discussed above. Thus, multiple mechanisms may concur in driving chronic immune activation and exhaustion of the naive CD4+ T cell pool in ICL.

TSLP responses were explored because this cytokine shows a degree of functional redundancy with IL-7 [34], [35], [54]. Mouse models have provided evidence for a contribution of TSLP to CD4^+^ T cell development, which becomes apparent only in the absence of IL-7 signaling [55]. TSLP also contributes to the recovery of the CD4^+^ T cell pool after subletal irradiation, as shown by a slower reconstitution in TSLPR knockout mice [55]. The role of TSLP in humans has been debated as freshly isolated human T cells, in contrast to those of mice, do not respond to TSLP stimulation [37], [41]. Activation through the TCR can confer a degree of TSLP responsiveness to human T cells maintained in culture, though the levels of STAT5 phosphorylation remain lower than those induced by IL-2 or IL-7 [37]. In contrast, TSLP powerfully activate human dendritic cells and primes them to induce Th2 responses, which is thought to promote allergic conditions [34], [35]. The detection of TSLP responses in CD4^+^ T cells of ICL patients suggests that TSLP does play a role in human T cell homeostasis. This is, to our knowledge, the first time that physiological TSLP responses have been detected in freshly isolated human T cells. The inverse correlation with IL-7 responses points to a compensatory role for TSLP, which may act as a second line homeostatic cytokine when the T cell pool is severely depleted. The frequency of TSLP-responsive cells remain relatively low in ICL, suggesting that TSLP responses alone are unlikely to fully compensate for the impairment of IL-7 responses, and possibly explaining why ICL patients remain lymphopenic. These observations are consistent with findings in mouse models, where TSLP functions become apparent in the absence of IL-7 signaling or in cases of lymphodepletion, but do not fully replace IL-7 functions. It will be important to determine whether TSLP responses are induced in other lymphopenic conditions, such as HIV infection or bone marrow transplantation.

Expression of the activation marker HLA-DR correlated with TSLP responses, suggesting that lymphopenia-driven immune activation increased the sensitivity of CD4^+^ T cells to TSLP. One mechanism is likely the upregulation of the TSLPR receptor chain, which was significant in CD4^+^ T cells of ICL patients, and which can be induced by TCR-dependent activation of human CD4^+^ T cells [37]. However, it should be noted that immune activation has an opposite influence on the second chain of the receptor, since it causes the internalization of IL-7Rα/CD127 [40], [44] These balancing influences may limit the amount of functional heterodimeric TSLP receptor, and may help explain why TSLPR expression did not simply correlate with TSLP responses. Immune activation may also promote TSLP responses at a step downstream of the receptor by facilitating the triggering of signaling, for instance through increased Jak1 and Jak2 activity [56], [57], or through the blockade of negative regulators of cytokine signaling such as SOCS proteins [58].

In conclusion, this study provides evidence for impaired IL-7 and IL-2 signaling in ICL, which likely contributes to the loss of CD4^+^ T cell homeostasis characteristic of this rare disease. The increase in TSLP responses reveals a new level of CD4^+^ T cell homeostatic regulation that may mitigate the effect of impaired responses to γc family cytokines. Understanding how cytokine networks are perturbed in ICL may further inform on fundamental mechanisms of CD4^+^ T cell homeostasis and may help devise immunotherapeutic approaches to restore CD4^+^ T cell counts and functions.

## Supporting Information

Figure S1 **Detection of cytokine receptors by flow cytometry.** Representative examples of staining for IL-2, IL-7, and TSLP receptor chains are shown for one healthy donor (HD, left column) and one ICL patient (ICL, middle column). Negative controls consisting in cells labeled with an isotype-matched irrelevant antibody (IgG1 or IgG2a), or without antibody (no-IgG) are shown in the right column. The plots are gated on the live CD3+ CD4+ CD45RA- memory population. (A) Staining for CD122/IL-2Rβ and CD25/IL-2Rα. (B) Staining for CD132/IL-2Rγ and CD25/IL-2Rα. (C) Staining for TSLPR and CD127/IL-7Rα.
(EPS)

Figure S2 **IL-7 responses in naive CD4+ T cells of ICL patients.** (A) Induction of pSTAT5 after stimulation with low dose IL-7. The inducible cytokine response (Δ%pSTAT5+) was measured by the difference in the percentage of pSTAT5+ cells before and after IL-7 stimulation in naive CD45RA+ CD4+ T cells. (B) Induction of pSTAT5 after stimulation with high dose IL-7. HD: healthy donors; ICL: patients with idiopathic CD4 lymphocytopenia. P values obtained with the Mann-Whitney U test are reported. (C and D) The percentage of IL-7-induced pSTAT5+ cells within the naive CD4+ T cell population is reported as a function of CD127 expression. (C) Responses to low dose IL-7 (D) Responses to high dose IL-7. (E) Correlation between pSTAT5 responses to high dose IL-7 stimulation in naive CD45RA+ CD4+ T cells and absolute CD4+ T cells counts in the blood of ICL patients. The Spearman correlation coefficient R and the corresponding P value are indicated.
(EPS)
